# Nucleoside Analogues for Chagas Disease and Leishmaniasis Therapy: Current Status and Future Perspectives

**DOI:** 10.3390/molecules29225234

**Published:** 2024-11-05

**Authors:** Emmanuel Awucha Nwoke, Silvester Lowe, Fawaz Aldabbagh, Karunakaran Kalesh, Hachemi Kadri

**Affiliations:** 1 Department of Pharmacy, School of Life Sciences, Pharmacy and Chemistry, Kingston University, Penrhyn Road, Kingston upon Thames, London KT1 2EE, UK; nwokeemmanuel7@gmail.com (E.A.N.); slyjlowe@gmail.com (S.L.); f.aldabbagh@kingston.ac.uk (F.A.); 2 School of Health and Life Sciences, Teesside University, Middlesbrough TS1 3BX, UK; k.karunakaran@tees.ac.uk

**Keywords:** nucleoside analogues, *Leishmania*, *T. cruzi*, NTDs, Trypanosoma, purine, pyrimidine

## Abstract

Chagas disease and leishmaniasis are two neglected tropical diseases that affect millions of people in low- and middle-income tropical countries. These diseases caused by protozoan parasites pose significant global health challenges, which have been exacerbated by the recent COVID-19 pandemic. There is an urgent need for novel therapeutics as current treatments are limited by toxicity and drug resistance. Nucleoside analogues, which have been extensively studied and successfully applied in antiviral and antitumor therapies, hold potential that has yet to be fully explored for treating these neglected diseases. In this review, we discuss the use of nucleoside analogues as promising therapeutic agents for Chagas disease and leishmaniasis. After briefly examining the pathology, progression, and current treatment options for these diseases, we provide a comprehensive analysis of the status of nucleoside analogues and explore their prospects. By outlining the current landscape and future directions, this review aims to guide research and development efforts towards more effective nucleoside-based treatments for Chagas disease and leishmaniasis.

## 1. Introduction

Neglected tropical diseases (NTDs) comprise a broad spectrum of twenty infectious diseases and conditions that predominantly affect the tropical and subtropical regions. These areas often suffer from limited access to clean water, poor healthcare infrastructure, and inadequate sanitation [1]. Despite causing significant morbidity and mortality, only 0.5% of drug candidates currently under development are directed towards NTDs [2]. The Global Observatory on Health Research and Development (R&D), an establishment of the World Health Organization (WHO), recently reported that a mere 0.6% of all health-related grant funding is allocated to the WHO’s list of NTDs [3]. The lack of funding and public awareness, as well as the recent COVID-19 pandemic, have further exacerbated the socioeconomic impact of these diseases on affected communities [4]. 

NTDs include diseases such as schistosomiasis, trachoma, dengue fever, lymphatic filariasis, African trypanosomiasis, Chagas disease, and leishmaniasis. Among these, Chagas disease and leishmaniasis are among the most neglected, with particularly high mortality rates in humans [5]. Efforts to combat these diseases through improved sanitation, healthcare, awareness, vector control, and mass drug administration have seen some success. However, the issues of drug resistance and toxicity necessitate ongoing research to develop new or alternative treatments [6]. 

### 1.1. Pathology and Progression of Chagas Disease

Chagas disease, also known as American trypanosomiasis, is caused by the protozoan parasite *Trypanosoma cruzi*. *T. cruzi* is a haemoflagellate intracellular parasite found mainly in endemic areas of 21 continental Latin American countries. The WHO estimates that over 12,000 annual deaths occur due to the clinical manifestations of Chagas disease [7]. The changing epidemiological profile and complex pathophysiology of Chagas disease have made it both an emerging disease and a significant global public health concern. 

The life cycle of *T. cruzi* occurs in three main stages: amastigote, epimastigote, and trypomastigote (the infective stage). *T. cruzi* is transmitted through the urine and faeces of infected insects belonging to the *Triatominae* family (kissing bugs). The bug feeds on animal or human blood and deposits its urine or faeces around the bite site. The parasite gains entry into the body as the individual smears the urine or faeces into the bite site, mouth, eyes, or any other skin break [8]. Contamination of food by these insects or their excrement can lead to foodborne epidemics of acute Chagas disease in humans that can occasionally be fatal. *T. cruzi* can also be transmitted through mother-to-child transmission, infected blood or blood products, and organ transplants [8]. 

Chagas disease manifests in two sequential phases: acute and chronic. The acute phase involves a high number of parasites circulating in the blood and lasts for about two months after infection [9]. Symptoms are often non-existent or mild, ranging from swelling around bite sites to fever, headaches, and swollen lymph nodes. Inadequately treated patients can progress into the chronic phase, where parasites are primarily concealed in the heart and gastrointestinal tract, causing the immediate symptoms to disappear. Between 10 and 30 years after the acute stage, long-term complications from the disease, such as heart failure (45% of infected individuals), enlarged colon and oesophagus (21% of infected individuals), and nerve damage (about 10% of infected individuals) may manifest following secondary immune suppression [9]. Unfortunately, this is often the stage at which most diagnoses are made.

### 1.2. Pathology and Progression of Leishmaniasis

Leishmaniasis is caused by *Leishmania* species parasites and is transmitted through the bite of infected female phlebotomine sandflies, with 350 million people at risk of infection due to poverty and lack of adequate housing infrastructure and healthcare. Thus, leishmaniasis remains a major public health issue [10].

The pathology of leishmaniasis is closely tied to the life cycle of the *Leishmania* parasite, which involves two primary stages: promastigote and amastigote. The process begins with the biting of the host (humans) by phlebotomine sandflies. Through phagocytosis, metacyclic promastigotes found in the infected sandflies gain entrance into macrophages, where they form phagosomes. The phagosome matures into a parasitophorous vacuole, where promastigotes transform into amastigotes. The infection spreads to either superficial or visceral cells and affects different tissues as amastigotes are released from ruptured macrophages [11]. 

Leishmaniasis in humans presents in three clinical forms: visceral, cutaneous, and mucocutaneous. Visceral leishmaniasis (VL) affects internal organs such as the spleen, liver, and bone marrow. It is prevalent in countries such as Ethiopia, Bangladesh, India, Brazil, Sudan, and South Sudan [10,11]. Cutaneous leishmaniasis (CL) is characterised by skin lesions, which can be self-healing but may leave significant scars. More than 75% of CL cases occur in Algeria, Colombia, Costa Rica, Peru, Sudan, Syria, and Brazil [10,11]. Mucocutaneous leishmaniasis (MCL) involves the mucous membranes of the nose, mouth, and throat, leading to disfiguring lesions. It is primarily found in regions like Thailand, Bolivia, Ethiopia, and Brazil [10,11].

### 1.3. Current Treatments for Chagas Disease and Leishmaniasis

**Figure 1:**
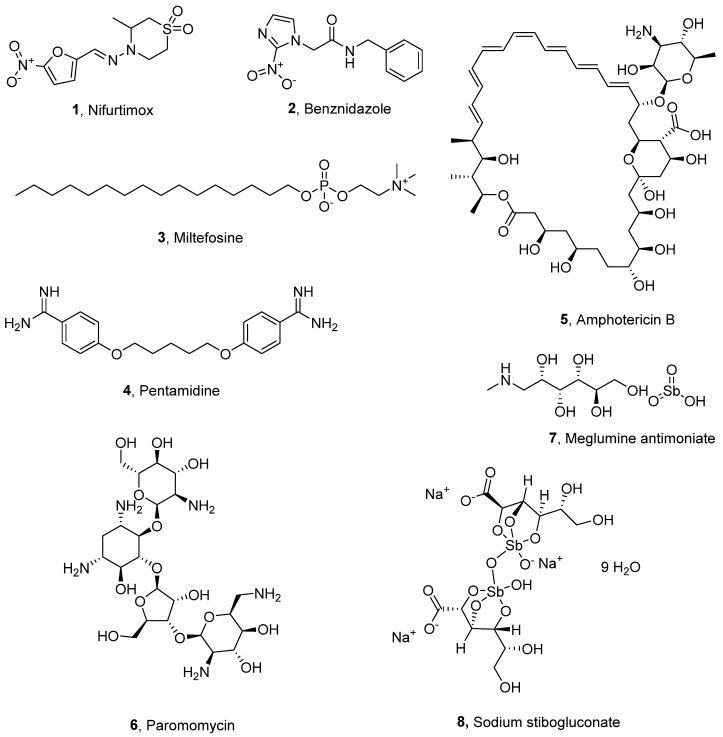
Current treatments for Chagas disease and leishmaniasis.

The primary treatments for Chagas disease are two non-nucleoside nitroheterocyclic prodrugs, nifurtimox (**1**) and benznidazole (**2**), which were discovered more than fifty years ago (Figure 1) [12]. Once inside the parasite, nitroreductases metabolise these drugs into antiparasitic electrophilic metabolites and reactive oxygen species [12]. While effective in the early stages of Chagas disease, their efficacy significantly decreases during the chronic stage. Additionally, prolonged use of these drugs can lead to severe side effects, resulting in poor treatment adherence in over 15% of patients [13,14]. 

In the case of leishmaniasis, drug treatments include miltefosine (**3**), pentamidine (**4**), amphotericin B (**5**), paromomycin (**6**), and pentavalent antimonies [14]. However, the primary treatment options remain as the antimonials meglumine (**7**) and sodium stibogluconate (**8**). Unfortunately, these treatments are associated with severe adverse effects of toxicity and the rapid development of drug resistance by the parasite [15,16].

The limitations of current treatments (above) and the pressing need for new therapeutic options have resulted in nucleoside analogues emerging as promising alternatives. This review examines various nucleoside analogues with demonstrated activity against Chagas disease and leishmaniasis and discusses the clinical prospects of this class of drugs. This review focuses on the activity of these compounds rather than the synthetic methodologies used to access nucleoside analogues. The synthesis and chemistry of nucleoside analogues have been extensively reviewed by others in the literature [17,18,19].

## 2. Nucleoside Analogues as Therapeutic Agents for Chagas Disease and Leishmaniasis

Nucleoside analogues have shown significant potential in the treatment of various diseases. These synthetic compounds are structurally like natural nucleosides and have been extensively studied for their antiviral and antitumoral properties [20]. Once inside the cell, nucleosides and their analogues undergo phosphorylation by nucleoside kinases, forming monophosphate metabolites (nucleotides). These nucleotides require two further phosphorylations to reach their active triphosphorylated form. The therapeutic efficacy of nucleoside analogues is largely due to the ability of this active form to be incorporated into DNA or RNA synthesis, disrupting key cellular processes, and eventually leading to cell death. 

Both *Leishmania* and *T. cruzi* are purine auxotrophs, meaning they are unable to synthesise purines endogenously and must rely on salvaging preformed purines from their host environment to meet their metabolic needs [21]. Hence, the purine salvage pathway (PSP) is a critical metabolic route for these parasites. In *Leishmania*, the PSP comprises extracellular nucleotidases that hydrolyse extracellular nucleotides into nucleosides, transporter systems that facilitate purine uptake across the cellular membrane, and catalytic enzymes that further process salvaged purines within the parasite. Similarly, in *T. cruzi*, this pathway involves essential enzymes like hypoxanthine-guanine phosphoribosyltransferase (HGPRT), which catalyse the formation of inosine monophosphate (IMP) and guanosine monophosphate (GMP) from the nucleobases hypoxanthine and guanine [21]. 

Targeting the PSP in these parasites using nucleoside analogues may offer a promising approach to disrupting their metabolism, potentially leading to the development of treatments for Chagas disease and leishmaniasis. 

### 2.1. Nucleosides with Anti-Chagas Disease Activity

**Figure 2:**
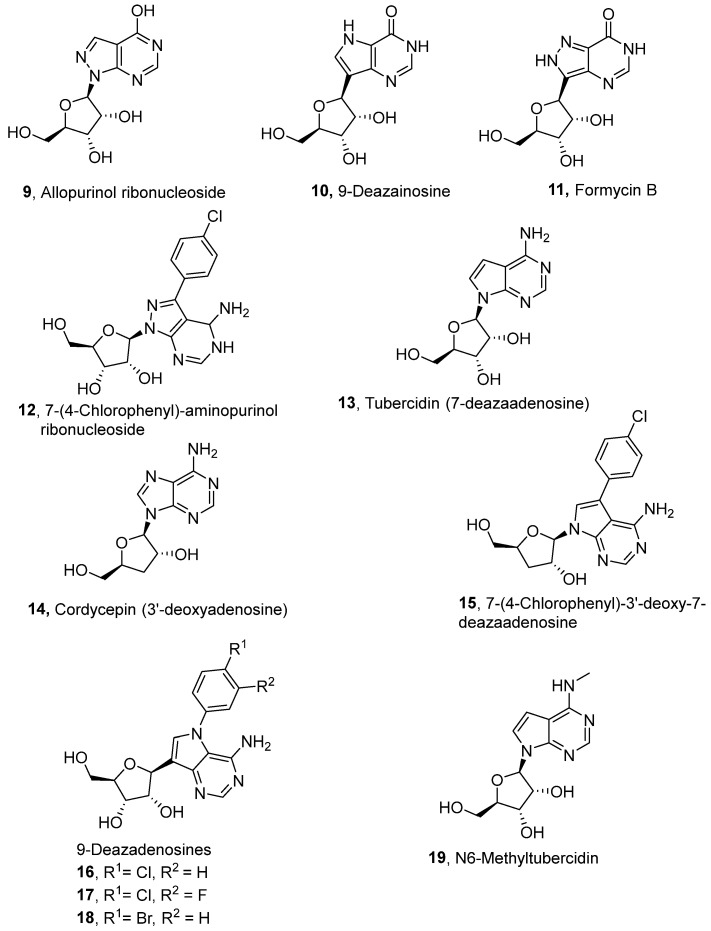
Nucleoside analogues with anti-Chagas disease activity.

Early attempts to use nucleoside analogues against Chagas disease included the use of inosines and their pyrazolopyrimidine analogues, particularly allopurinol (1*H*-pyrazolo [3,4-*d*]pyrimidin-4-ol), which can also be coupled with ribonucleosides in cells to form allopurinol ribonucleoside (4-hydroxypyrazolo[3,4-*d*]pyrimidine ribonucleoside (**9**)) (Figure 2) [22]. In 1984, Marr et al. investigated the biological action of inosine-based nucleoside analogues against *L. donovani* and *Trypanosoma* species [22]. The study systematically modified the five-membered heterocyclic ring of six inosine analogues to assess their antiprotozoal activities and toxicity against a mammalian cell line. All six inosine analogues showed activity against *T. cruzi* and *L. donovani* species, displaying 50% effective doses in the range of 1 to 10 µM. However, only allopurinol ribonucleoside (**9**), 9-deazainosine (**10**), and formycin B (**11**) exhibited low toxicity against the mouse L cells [22]. A year later, this finding was supported in a study by McCabe et al. with the organism at concentrations as low as 0.1 µg/mL, whereby formycin B (**11**) inhibited the in vitro intracellular replication of *T. cruzi* and protected mice from death caused by acute infection [23].

More recently, Bouton et al. synthesised several 7-substituted and ribofuranose-modified pyrazolo[3,4-*d*]pyrimidine nucleosides, which included both adenosine-like (aminopurinol riboside) and inosine-like (allopurinol riboside) analogues for assessment against *T. cruzi* and *L. donovani* [24]. 7-(4-chlorophenyl)-aminopurinol ribonucleoside (**12**) showed the highest anti-trypanosomal activity (IC_50_ = 0.32 μM) of all evaluated analogues. Structure–activity testing suggested that the chloro-substituent not only reduced the electron density of the phenyl ring but was likely involved in a crucial interaction between the molecule and a parasitic target or transporter [24].

Purine nucleoside analogues of natural products with anti-*T. cruzi* activities have been explored. Tubercidin (7-deazaadenosine; **13**) is an antibiotic and adenosine analogue that was extracted from *Streptomyces tubercidicus* by Anzai et al. [25]. Tubercidin is active against *T. cruzi*, and efforts have been made to optimise its potency [26]. It was necessary to find safer analogues for tubercidin that, nonetheless, had antiparasitic properties because tubercidin was too toxic to use against Chagas disease. Cordycepin, the 3′-deoxyadenosine analogue (**14**), also has anti-trypanosomal activity [26]. Hulpia et al. synthesised 33 nucleoside analogues based on a structural scaffold that combined the 3′-deoxyribose sugar moiety of cordycepin with differently 7-substituted 7-deazaadenine bases. [26]. The in vitro potency of these analogues was evaluated against intracellular *T. cruzi* amastigotes as well as for cytotoxicity against MRC-5 fibroblasts. One of the analogues, **15**, displayed potent cellular activity and in vivo evaluation in an acute infection murine model of *T. cruzi* and displayed 94.6% parasitemia reduction on a 5-day oral-dosing twice-daily schedule without apparent toxicity [26].

Based on these initial discoveries, different analogues have been made for tubercidin. Of particular interest is the replacement of the C-N glycosidic bond with a C-C bond. *C*-nucleosides have proven to be more stable by being less acid-labile and less prone to enzymatic cleavage, unlike *N*-nucleosides [27,28]. Bouton et al. synthesised *C*-nucleosides of tubercidin lead compounds with the expectation of enhancing their anti-trypanosomal activity [29]. Of these *C*-nucleosides, three N9 phenyl compounds (**16**, **17**, and **18**) displayed potent anti-*T. cruzi* activity at low to submicromolar concentrations with a favourable cytotoxicity profile. These findings indicated that modifying the nucleobase moiety can sustain the potency of the nucleoside analogues while enhancing their selectivity [29].

Lin et al. adopted a different strategy focusing on the modification of the 6-amino group of tubercidin and synthesised 32 analogues for in vitro and in vivo anti-trypanosomal activities [30]. The in vitro evaluation against intracellular amastigotes of *T. cruzi* and *L. infantum* showed that the 6-methylamino analogue (**19**) displayed the most potent activity against both parasites. Compound **19** exhibited submicromolar potency and high selectivity against the parasites, with no cytotoxicity observed for the host cells (*T. cruzi* EC_50_/7 days = 0.45 µM and *L. infantum* EC_50_/5 days = 0.49 µM) [30]. In vivo evaluation of this compound in an acute Chagas disease mouse model demonstrated dose-dependent parasite load reductions, achieving a significant reduction in peak parasitemia levels and increased animal survival rates at 12.5 mg/kg/day. However, higher doses led to acute toxicity and weight loss in mice [30].

Barnados-Carceller et al. evaluated the capacity of 23 purine analogues with different substitutions in the complementary chains of their purine rings for the inhibition of the growth of *T. cruzi* [31]. The level of toxicity in HepG2 and Vero cells and the capacity to inhibit the development of amastigotes were examined. Eight of the tested compounds showed specificity against the test organism (with an IC_50_ between 2.42 and 8.16 µM). Using in silico docking, the most likely targets were determined as hypoxanthine-guanine phosphoribosyl transferase and adenine phosphoribosyl transferase [31].

### 2.2. Nucleosides with Anti-Leishmanial Activity

**Figure 3:**
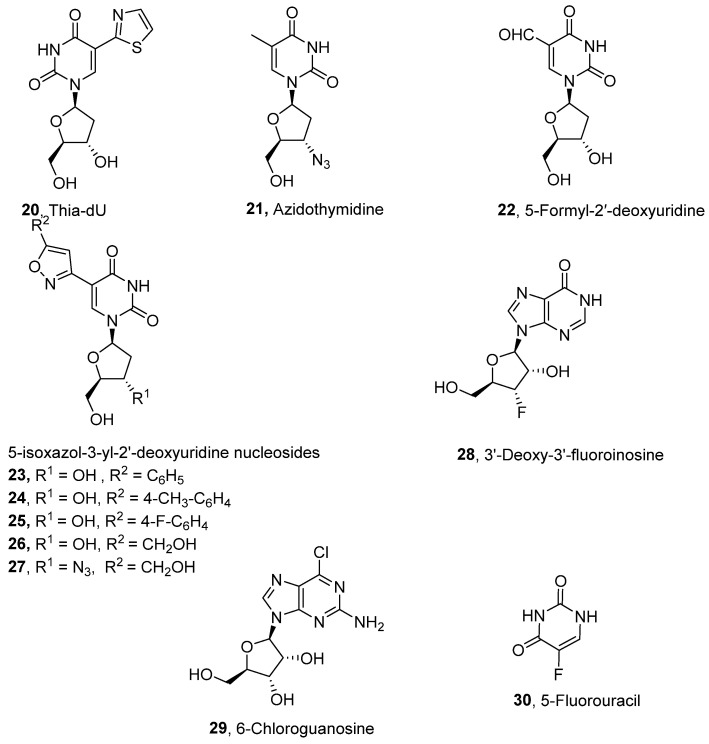
Nucleoside analogues with anti-leishmanial activity.

Pyrimidine nucleosides have been reported to exhibit activity against *Leishmania* species. In 2005, Peyron et al. synthesised and evaluated in vitro eight 5-substituted 2′-deoxyuridine nucleosides against *L. donovani* promastigotes and amastigotes [32]. The most active compound was Thia-dU (**20**) with an IC_50_ of 3 μM against *L. donovani* promastigotes with activity comparable to that of the antiviral azidothymidine (**21**), which was used as a reference (IC_50_ = 3.1 μM) (Figure 3) [32]. Based on this discovery, a year later, Torrence and coworkers explored the ability of 5-substituted pyrimidine nucleosides to inhibit *L. donovani* growth in culture [33]. Of the 31 synthesised compounds, 5-formyl-2′-deoxyuridine (**22**), with an IC_50_ of 0.9 μM, was three times more active than azidothymidine [33].

To simplify the synthetic approaches in preparing these pyrimidine-based nucleosides, Guo et al. developed a more efficient synthetic route for C5-(isoxazol-3-yl)-pyrimidine nucleosides [34]. Through this approach, 30 analogues were prepared and tested in vitro for their anti-leishmanial activities against *L. donovani* intramacrophage amastigotes. Many compounds (**23**, **24**, **25**, **26**, and **27**) showed potent anti-leishmanial activity in the submicromolar range [34]. Notably, the activity of these compounds was potentially linked to the parasite’s pyrimidine/purine transporter since the –OHs free nucleoside derivatives were mostly more active than the acetyl-protected counterparts [34]. 3′-FI was effective against L. donovani amastigotes in J774.1 cells in an in vitro cultivation system under conditions like those used in the in vivo assay.

In addition to the earlier mentioned inosine-based compounds with anti-leishmanial activity (**9**, **10**, **11**, and **19**; Figure 2), more inosine nucleosides have been evaluated in vitro for anti-leishmanial activity. 3′-deoxy-3′-fluoroinosine (3′-FI) (**28**; Figure 3) was shown to exhibit robust anti-leishmanial activity by producing EC_50_ values of 2.3 × 10^−7^ and 1.0 × 10^−6^ M against the promastigotes of *L. tropica* and *L. donovani,* respectively [35]. The study also showed that 3′-FI was less toxic to the model host (mouse mammary tumour FM3A cells) due to its metabolism from 3′-FI to 3′-deoxy3′-fluoroadenosine 5′-triphosphate (3′-FATP) within *Leishmania* promastigotes, a process that does not occur within FM3A cells. When tested at 50 mg/kg intravenously, a similar cytotoxic effect against *L. donovani* amastigotes in mice was exhibited by 3′-FI [35].

Tran et al. developed a series of forty-seven adenosine analogues with modifications at the 2-, 5-, and 6′-positions and three 7-deazaguanosine derivatives designed to serve as selective substrates for protozoan nucleoside salvage enzymes while remaining ineffective in mammalian cells [33]. Of these compounds, a modified guanosine (**29**) displayed potent activity against *L. donovani* in vitro (IC_50_ = 60 nM; SI = 2720) and was more active than miltefosine (IC_50_ = 122 nM) [36].

Indeed, the pyrimidine salvage pathway is a very promising target in the chemotherapy of leishmaniasis [37]. Azzouz et al. investigated the inhibitory impact of pyrimidine analogues; cytarabine and 5-fluorouracil and purine analogues; and azathioprine and 6-mercaptopurine on the cellular growth of promastigotes and the intracellular amastigote stages of *L. donovani* and *L. infantum* [38]. The anticancer compound 5-fluorouracil (**30**), which is converted in vivo into the active metabolite 5-fluoroxyuridine monophosphate (F-UMP), was the only compound effective against both stages [38].

Uridine-based carbocyclic nucleosides with a methylene group replaced by the oxygen atom in the furanose sugar moiety are more stable to enzymatic cleavage since the hemi-aminal of the glycosidic bond is converted to a tertiary amine [39]. The first naturally occurring carbocyclic nucleosides were neplanocin A and aristeromycin, which exhibited high antiviral activity [40]. However, due to the close resemblance of their triphosphate forms to ATP, they also exhibited significant toxicities. This challenge can be overcome by removing the CH_2_ group of the 5′-hydroxymethyl, resulting in what are known as 5′-norcarbocyclic nucleosides [40]. The truncated nature of the 5′-hydroxyl groups is not recognised and phosphorylated by kinases, and they are, thus, not converted to their corresponding triphosphates [37]. Alzahrani et al. designed and synthesised 5′-norcarbocyclic pyrimidine nucleosides analogues as potential anti-*Leishmania* agents. Of these analogues, the 4′,N^3^-di-(3,5-dimethylbenzoyl)-substituted analogues showed the most prominent activity in an in vitro evaluation against *Leishmania mexicana* but in the low micromolar range [41].

All the nucleoside analogues covered in this review and their documented in vitro activity against *T. cruzi* and *Leishmania* species are outlined in Table 1.

## 3. Nucleoside Analogues in the Clinic with Anti-Leishmanial and Anti-Chagas Disease Activity

**Figure 4:**
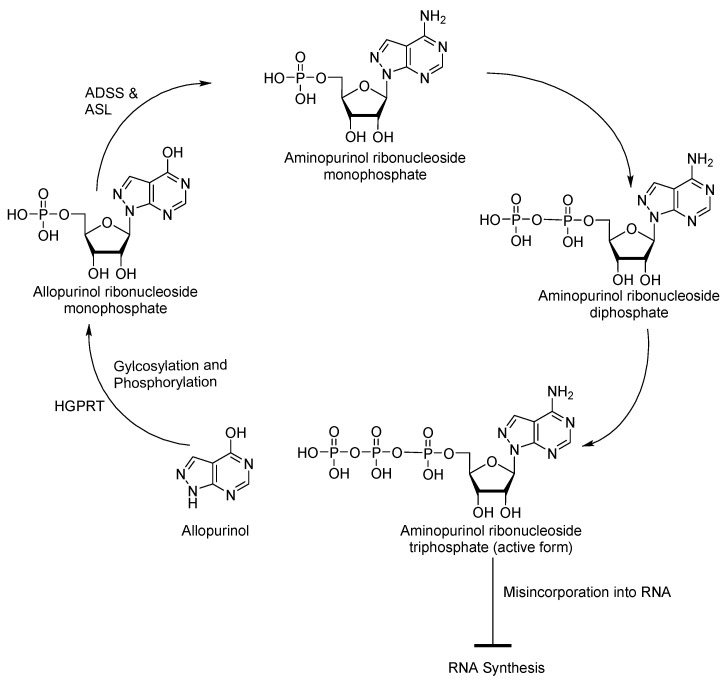
Mechanism of action of allopurinol in *Leishmania* and *T. cruzi*.

Despite the various nucleoside analogues with anti-Chagas disease and anti-leishmanial activity discussed above, there are limited reports on their use in clinical settings, except for limited studies on the purine analogue allopurinol, which is converted intracellularly into allopurinol ribonucleoside [42,43]. The metabolism and activity of allopurinol in *T. cruzi* and *L. donovani* have been investigated, and its proposed mechanism of action is illustrated in Figure 4 [44,45,46]. Once inside the parasite, HGPRT transfers a 5-phosphoribosyl group from 5-phosphoribosyl-1-pyrophosphate to allopurinol, converting it into allopurinol ribonucleoside monophosphate, an inosine-like derivative. Allopurinol ribonucleoside is then converted into the adenosine-like derivative aminopurinol ribonucleoside monophosphate by adenylosuccinate synthase (ADSS) and adenylosuccinate lyase (ASL). The double phosphorylation of aminopurinol ribonucleoside monophosphate results in its activation and misincorporation into RNA, resulting in the disruption of RNA synthesis and, consequently, blocking protein synthesis. As mentioned above, this metabolic pathway is particularly important for *Leishmania* and *T. cruzi*, as they lack the ability to synthesise purines de novo and, thus, rely on salvaging preformed purines from their host environment [21].

Although allopurinol has demonstrated effectiveness in vitro, and its mechanism of action is well elucidated, the few clinical trials conducted have produced inconclusive results. Kager et al. used allopurinol in the treatment of 10 patients with visceral leishmaniasis [42]. Allopurinol was administered in oral doses ranging from 16 mg/kg/day to 24 mg/kg/day divided into three daily doses. While satisfactory results were achieved in four out of six patients who had previously failed to respond to Pentostam (sodium stibogluconate, compound **8**; Figure 1), the response in four treatment-naïve patients was less satisfactory. One of the ten patients experienced a relapse of VL, suggesting the need for continued therapy for a longer period, even after a parasitological cure [42]. Rassi et al. investigated the effectiveness of allopurinol as a treatment for chronic Chagas disease. In their study, Rassi et al. conducted a double-blind clinical trial involving 35 individuals from endemic areas of Central Brazil, all of whom were in the chronic phase of Chagas disease [43]. The participants were randomly assigned to receive either allopurinol (900 mg/day) or a placebo for a duration of 60 days. The results indicated that allopurinol at the doses used did not significantly reduce parasitemia or improve clinical outcomes compared with the placebo group [43].

The need for higher doses of allopurinol and prolonged therapy could be explained by the intricate host–parasite interactions and implications on the pharmacokinetics of drugs used to treat these parasites [47,48]. Indeed, although recent studies have explored the pharmacokinetic and pharmacodynamic relationships in VL, our understanding of the parasite distribution within the host, especially for Chagas disease, remains limited [49,50].

## 4. Future Directions

Given the socioeconomic context of populations affected by these neglected diseases, developing cost-effective medicines and ensuring treatment accessibility should remain top priorities. The WHO’s target product profile directory proposes that when developing treatments for Chagas disease and leishmaniasis, the focus should be on oral, age-adapted, short-course therapies that are safe, effective, and affordable [51]. These treatments should be stable in harsh conditions and accessible, especially in low-resource settings, including for vulnerable populations like pregnant women and immunocompromised patients [51]. One approach to achieving this is drug repurposing, a promising strategy for accelerating drug discovery and development. By identifying new therapeutic applications for existing drugs, this approach can significantly reduce costs and time to market. As highlighted above, some limited attempts have been made to repurpose allopurinol, a xanthine oxidase inhibitor used to treat gout, for treating leishmaniasis and Chagas disease [42,43]. Similarly, other antiviral nucleosides currently in use could be investigated for potential repurposing for leishmaniasis and Chagas disease. Utilising computational tools to predict drug repurposing candidates can accelerate this process [52,53].

The inherent pharmacokinetic issues of nucleoside analogues present a primary challenge that can significantly impact their clinical effectiveness [54,55]. For example, cordycepin encounters notable pharmacokinetic limitations primarily due to its rapid metabolism. Studies indicate that cordycepin is quickly deaminated by adenosine deaminase in vivo, which significantly shortens its half-life and limits its bioavailability [55]. This rapid degradation results in a substantial decrease in plasma levels shortly after administration, making it challenging to achieve effective therapeutic concentrations [55]. However, structural modifications to generate focused nucleoside analogue libraries may address these limitations by producing derivatives with better pharmacokinetic profiles. Hulpia et al. demonstrated the success of this approach by combining the molecular scaffolds of cordycepin and tubercidin to develop 3′-deoxytubercidin as a potent and orally bioavailable chemotherapeutic candidate for treating human African trypanosomiasis, which is caused by another kinetoplastid parasite, *Trypanosoma brucei* [56]. Prodrug strategies may also offer a promising avenue to improve the bioavailability and phosphorylation efficiency of nucleoside analogues. Advancements in prodrug technologies, such as the clinically proven ProTide technology, have facilitated the development of nucleoside analogue monophosphates, enhancing drug delivery and efficacy [57,58]. Such prodrug strategies can improve the pharmacokinetic properties and bioavailability of nucleoside analogues, making them more effective in combating these infectious diseases.

Additionally, combination therapies involving nucleoside analogues have been explored in various diseases, such as HIV, hepatitis B, and cancer, demonstrating improved outcomes compared with single-agent therapies [59]. Therefore, combination therapies exploring synergies between nucleoside analogues and existing antiparasitic drugs may offer improved treatment outcomes and reduced long-term side effects. A recent example is a study by Mazzeti et al., which evaluated the effect of allopurinol combined with nitroheterocyclic compounds (benznidazole and nifurtimox) on infection with a strain of *T. cruzi*. The administration of these drugs in combination exerted synergistic effects, with some combinations achieving a 100% cure in infected animals [60]. Present et al. investigated the use of an *N*-methyl tubercidin analogue (CL5564) in a mouse model of CL infection and found out that two-thirds of the treated animals reached sterile cure only when used in combination with miltefosine [61]. Fiuza et al., in a phenotypic evaluation of nucleoside analogues against *T. cruzi* infection, concluded that combination with nitro drugs is needed to identify more effective and safer therapies for Chagas disease [62].

Finally, given the complexity of these diseases and the challenges inherent to drug development, particularly for NTDs, collaborative efforts are essential for discovering potent nucleoside analogues to treat leishmaniasis and Chagas disease. Global initiatives like the Drugs for Neglected Diseases Initiative (DNDi)’s recent development of the pyrrolopyrimidine derivative DNDI-6174, which inhibits the *Leishmania* cytochrome bc1 complex (III), exemplify the success of such collaborations [63]. DNDI-6174 has met all target candidate profile criteria required to advance into preclinical development, highlighting the importance of joint efforts in advancing potential treatments for these neglected tropical diseases [63].

## 5. Conclusions

Overall, despite ongoing efforts to use nucleoside analogues for the treatment of Chagas disease and leishmaniasis, their full therapeutic potential has yet to be realised. The exploration of this class of drugs for these diseases has revealed both promising opportunities and significant challenges. Encouragingly, recent advancements in drug discovery and prodrug technologies are driving progress towards more effective nucleoside-based therapies. Notably, repurposing existing nucleoside analogues and exploring combination therapies hold promise for improving treatment outcomes and reducing side effects. However, collaborative efforts and the integration of multidisciplinary approaches are essential for overcoming current challenges. These efforts will be crucial to translating laboratory successes into clinical applications, ultimately alleviating the burden of these neglected diseases on affected communities.

## Figures and Tables

**Table 1:** Summary of nucleoside analogues with anti-Chagas disease and anti-leishmanial activities.

Compound (Number)	In Vitro Activity (IC_50_ μM)			Structure	Reference
	*T. cruzi*	*L. donovani*	*L. infantum*		
Allopurinol ribonucleoside (**9**)	2–10	7		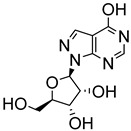	[22]
9-deazainosine (**10**)	1	1		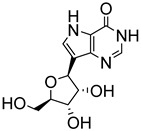	[22]
Formycin B (**11**)	0.5	1		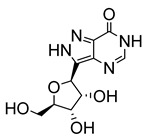	[22,23]
7-(4-chlorophenyl)-aminopurinol ribonucleoside (**12**)	0.32		>64.0	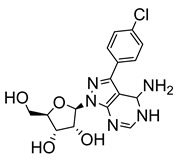	[24]
Tubercidin (7-deazaadenosine) (**13**)	0.34		0.13	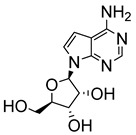	[25]
Cordycepin (3′-deoxyadenosine) (**14**)	2.51			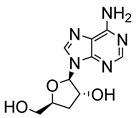	[26]
7-(4-chlorophenyl)-3′-deoxy-7-deazaadenosine (**15**)	0.047			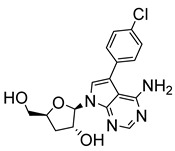	[26]
9-deazadenosines (**16**) R_1_= Cl; R_2_ = H (**17**) R_1_= Cl; R_2_ = F (**18**) R_1_= Br; R_2_ = H	0.34 1.04 0.46		>64.0 >64.0 >64.0	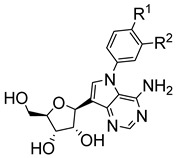	[29]
N6-methyltubercidin (**19**)	0.45		0.49	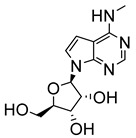	[30]
Thia-dU (**20**)		3.1		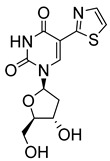	[32]
Azidothymidine (**21**)		3.1		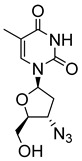	[32]
5-formyl-2′-deoxyuridine (**22**)		0.9		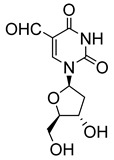	[33]
5-isoxazol-3-yl-2′-deoxyuridine nucleosides				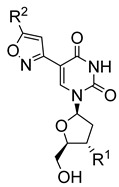	
(**23**) R^1^ = OH; R^2^ = C_6_H_5_		0.76			
(**24**) R^1^ = OH; R^2^ = 4-CH_3_-C_6_H_4_		0.61			
(**25**) R^1^ = OH; R^2^ = 4-F-C_6_H_4_		0.58			[34]
(**26**) R^1^ = OH; R^2^ = CH_2_OH		0.73			
(**27**) R^1^ = N_3_; R^2^ = CH_2_OH		0.49			
3′-deoxy-3′-fluoroinosine (**28**)		1		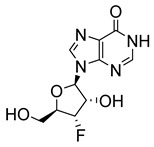	[35]
6-chloroguanosine (**29**)		0.06		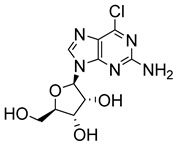	[36]
5-fluorouracil (**30**)		1	3	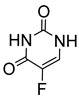	[38]

## Data Availability

Data sharing is not applicable. No new data were created or analyzed in this study.
